# Powerful Inhibition of Experimental Human Pancreatic Cancers by Receptor Targeted Cytotoxic LH-RH analog AEZS-108

**DOI:** 10.18632/oncotarget.1044

**Published:** 2013-06-03

**Authors:** Karoly Szepeshazi, Andrew V. Schally, Norman L. Block, Gabor Halmos, Mehrdad Nadji, Luca Szalontay, Irving Vidaurre, Andrew Abi-Chaker, Ferenc G. Rick

**Affiliations:** ^1^ Veterans Affairs Medical Center, Miami, FL; ^2^ South Florida VA Foundation for Research and Education, Miami, FL; ^3^ Department of Pathology, University of Miami, Miller School of Medicine, Miami, FL; ^4^ Division of Hematology/Oncology, University of Miami, Miller School of Medicine, Miami, FL; ^5^ Division of Endocrinology, Department of Medicine, University of Miami, Miller School of Medicine, Miami, FL; ^6^ Department of Biopharmacy, School of Pharmacy, University of Debrecen, Hungary; ^7^ Department of Urology, Florida International University, Herbert Wertheim College of Medicine, Miami, FL

**Keywords:** pancreatic carcinoma, targeted therapy, LH-RH receptor, cytotoxic LHRH analog, GnRH, doxorubicin, peptide therapy

## Abstract

Pancreatic carcinoma is one of the cancers with the worse prognosis, thus any therapeutic improvement is imperative. Cytotoxic LH-RH analog, AN-152 (proprietary designation, AEZS-108), consisting of doxorubicin (DOX) conjugated to D-Lys^6^LH-RH, is now in clinical trials for targeted therapy of several sex hormone-dependent tumors that express LH-RH receptors. We investigated LH-RH receptors in human pancreatic carcinoma and the effects of AN-152 (AEZS-108) on experimental pancreatic cancers. We determined LH-RH receptor presence in human pancreatic cancer samples by immunohistochemistry and, in three human pancreatic cancer lines (SW-1990, Panc-1 and CFPAC-1), by binding assays and Western blotting. The effects of the cytotoxic LH-RH analog were investigated on growth of these same cancer lines xenografted into nude mice. We also analyzed differences between the antitumor effects of the cytotoxic analog and its cytotoxic radical alone, doxorubicin (DOX), on the expression of cancer-related genes by PCR arrays. LH-RH receptors were expressed in two randomly selected surgically removed human pancreatic cancer samples and in all three cancer lines. Cytotoxic LH-RH analogs powerfully inhibited growth of all three tumor lines in nude mice; AN-152 was significantly stronger than DOX on Panc-1 and CFPAC-1 cancers. PCR array showed that cytotoxic LH-RH analog AN-152 affected the expression of genes associated with cellular migration, invasion, metastasis and angiogenesis more favorably than DOX, however the changes in gene expression varied considerably among the three cancer lines. Cytotoxic LH-RH analog, AEZS-108, may be a useful agent for the treatment of LH-RH receptor positive advanced pancreatic carcinoma.

## INTRODUCTION

Pancreatic carcinoma is the 9^th^ most common human cancer in the Western world, including the USA, but it is the fourth leading cause of cancer death in both males and females. [1] Its incidence has increased in the white population by about 1% per year between 1999 through 2008. [2] Ductal carcinomas of the pancreas are very aggressive tumors, and because of the concealed location of the gland, they are mostly detected in late stages of development when surgical removal is not useful. Thus, the 5-year survival rate is below 5% and the median survival time after diagnosis is less than 6 months. [3] There is not an accepted uniform standard therapy around the world for pancreatic carcinoma; treatment methods vary by location and economic circumstances. [4] Current treatment modalities such as gemcitabine alone or in combination with 5-fluorouracil, radiation or erlotinib, (an EGF receptor tyrosine kinase inhibitor), provide minimal palliation for patients with advanced pancreatic cancers; latest reviews from around the world express overall disappointment with these results. [5-8] Recent trials showed that a combination regimen, FOLFIRINOX (5-fluorouracil, leucovorin, irinotecan and oxiplatin), showed superiority to gemcitabine monotherapy, but the combination is highly toxic and is recommended only for fit patients. [9-11] On the other hand, most publications express the opinion, so far justified, that the rapidly growing body of information on the molecular pathogenesis of pancreatic cancer will provide new therapeutic approaches for these patients. Because of the poor survival rates and the low response rates to chemotherapy, the NCI suggests that “Clinical trials are appropriate alternatives for treatment of patients with any stage of disease and should be considered prior to selecting palliative approaches”. [12]

One possible way to increase the efficacy of therapy is to target drugs to specific molecular components in cancer cells, such as growth factors and their receptors, or proliferation-promoting kinases etc. [13-16] Specific receptors for hypothalamic peptide hormones, including LH-RH, somatostatin and bombesin have been detected in various cancers, and these receptors may serve as targets for the peptide ligand linked to a cytotoxic agent. [13, 15, 17-21] Thus we have developed new classes of targeted antitumor agents by linking doxorubicin (DOX), or its derivative, 2-pyrrolino-DOX (AN-201), to LH-RH, somatostatin and bombesin. Since these cytotoxic compounds are targeted to receptors for these specific hormones on tumor cells, the concentration of the toxic agent in tumor tissue is increased and normal cells are spared from exposure to toxicity. We demonstrated that in animal models, cytotoxic analogs of LH-RH, somatostatin and bombesin may each have very powerful inhibitory effects on cancers that express the corresponding receptors for the carrier peptide; these hybrid compounds are more effective and less toxic than the cytotoxic radicals alone. [13, 15, 21]

Based on information that receptors for somatostatin and bombesin are expressed in pancreatic cancers we tested the effects of cytotoxic analogs of somatostatin (AN-238) and bombesin (AN-215) on human pancreatic cancer lines xenografted into nude mice. AN-238 and AN-215 significantly inhibited growth of SW-1990, Panc-1, CFPAC-1, Capan-1 and Capan-2 tumors each of which expresses various subtypes of somatostatin receptors and bombesin receptors. [22, 23] LH-RH and its receptor are not confined to the hypothalamic-pituitary axis. [24] In the periphery, the LH-RH system coordinates gonadal functions and serves as a growth factor of benign conditions [25-28] and various malignancies. [29, 30] Following our first demonstration of LH-RH receptors in pancreatic cancers in 1989, several other groups have confirmed that receptors for LH-RH are present in pancreatic tumors [15, 21] Based on these findings, in the present study, we investigated the effects of cytotoxic LH-RH analog AN-152 (AEZS-108), consisting of [D-Lys^6^]LH-RH linked to DOX-14-*O*-hemiglutarate on various human pancreatic cancers in nude mice. [13] This analog has had strong inhibitory effects on experimental prostate, breast, ovarian, endometrial, renal and colorectal carcinomas, as well as non-Hodgkin's lymphomas and melanomas. [13, 15, 21] AN-152 (AEZS-108), is now in phase I/II and III clinical trials for the treatment of human ovarian, endometrial, prostate and bladder cancers. [31]

## RESULTS

### LH-RH receptor expression in clinical pancreatic cancer specimens and in human cancer lines

**Figure 1:**
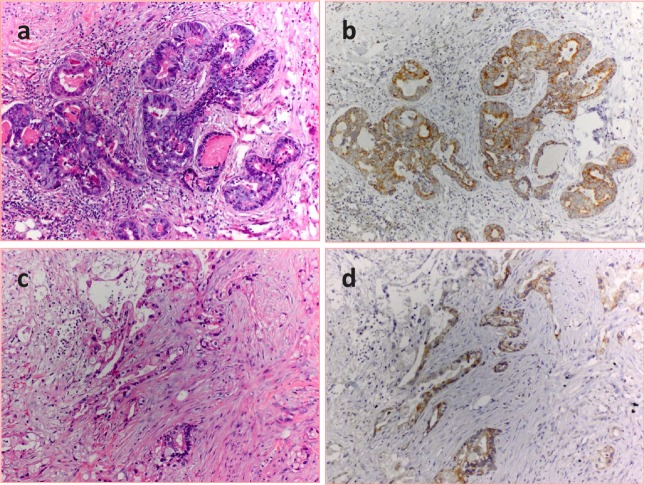
Expression of LH-RH receptors in human pancreatic cancer Sections of two randomly selected carcinomas were stained with hematoxylin-eosin (a and c) and LH-RH receptors were detected in parallel sections with immunohistochemistry as described in the Materials and Methods. The receptors appear in brown in the cancerous glandular epithelia (b and d).

Immunohistochemical investigation verified the expression of LH-RH receptors in two randomly selected archived samples of surgically removed human pancreatic cancers (Fig. 1). Positive staining was observed in the malignant epithelial cells, but not in stromal elements or peritumoral tissues.

In untreated samples of the three human pancreatic cancer lines, high affinity and low capacity LH-RH receptors were detected. The dissociation constants and maximal binding capacities of the receptors were as follows: SW-1990: K_d_= 4.51±0.51 nM, B_max_=624.3±24.3 fmol/mg protein; Panc-1: K_d_=6.27±0.08 nM, B_max_=495.3±17.4 fmol/mg membrane protein; CFPAC-1: K_d_=3.89±0.12 nM, B_max_=306.0±27.5 fmol/mg protein.

**Figure 2:**
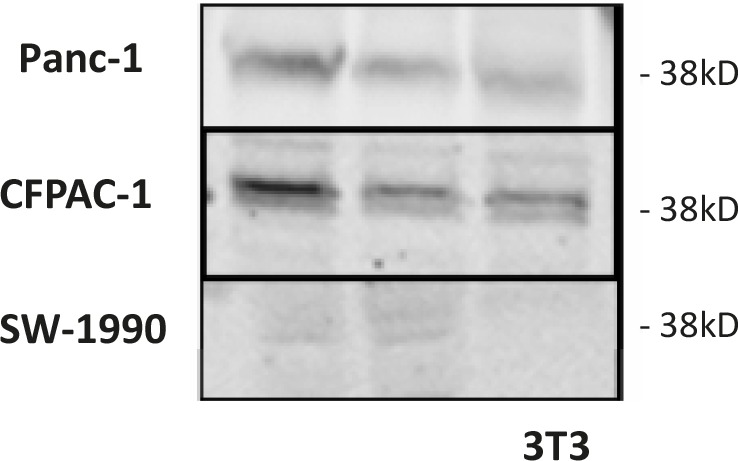
LH-RH receptor protein (38 kD) was detected by Western blotting in three human pancreatic cancer lines grown in nude mice Representative blots of three independent experiments are shown. The lower right corner shows proteins from 3T3 cells as negative control.

LH-RH receptor protein (38 kD), detected with Western blotting, was present in all three tumor lines; the amount of this protein was not significantly different between treated and control samples (Fig. 2)

Earlier binding studies demonstrated moderate-affinity binding sites for LH-RH also in nitrosamine-induced pancreatic cancers in hamsters (13).

### Effect of treatment on tumor growth in nude mice

**Figure 3:**
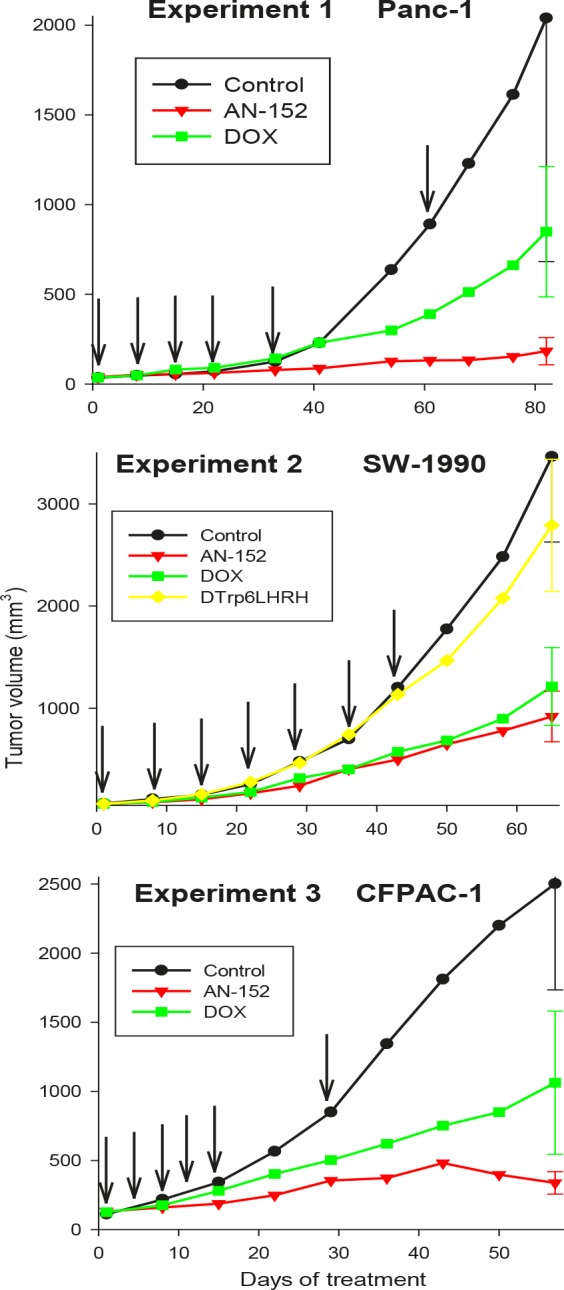
Effect of treatment with cytotoxic LH-RH analog, AN-152 (AEZS-108), and its cytotoxic radicals, doxorubicin (DOX), on growth of human pancreatic cancers in nude mice The vertical bars represent SE. Arrows show days of treatment.

In Experiment 1, AN-152 powerfully inhibited growth of Panc-1 cancers in nude mice, while DOX had only a moderate but not significant inhibitory effect (Fig. 3, Table 1). Neither treatment had an effect on body and organ weights of the mice.

**Table 1:** Effect of treatment with cytotoxic LH-RH analog AN-152 on volume and weights of human pancreatic cancers in nude mice

Experiments/Groups	Tumor volume (final) (mm3)	Tumor weights (mg)	
Panc-1			
Control	2040±1357	606±340d	
AN-152	183±76a	93±161a	
DOX	849±363	689±139	
2. SW-1990		Tumors with cysts Solid tumor part Cystic/solid tumor	
Control	3464±838	3729±921 2473±636 1.56±0.12	
AN-152	919±248a	1160±288a 371±69a 2.58±0.42b	
DOX	1213±381a	1496±466 585±141a 1.93±0.30	
4. D-Trp6[LH-RH]	2791±646	3107±696 1862±361 1.56±0.11	
3.CFPAC-1			
Control	2502±769	518±244c	
AN-152	338±81a	360±102	
DOX	1063±518	540±140	

Values are means ± SE aP<0.05 vs. Control bP<0.05 vs. all other groups cSome tumors in these experiments contained cysts and were weighed after cysts were cut and fluid released.

In Experiment 2, cysts containing clear yellowish or brownish liquid developed in many tumors SW-1990 starting on treatment day 30. These cysts grew very rapidly, making tumor volume results non-germane. Thus the volumes of macroscopically cystic and non-cystic tumors were calculated separately. Similarly, at autopsy, the cystic tumors were weighed, the tumors cut, and the fluid released, and the solid parts weighed again. Tumor volume changes are shown in Figure 3, the numerical data are shown in Table 1. Tumor volume was significantly lower in both groups treated with cytotoxic compounds. Weights of cystic tumor were significantly reduced by treatment with AN-152 and solid tumor weights were reduced by AN-152 and DOX. Weight ratio of cystic versus solid tumors was highest in the groups receiving AN-152 showing that the fluid amount was significantly larger in this group than in the other groups. Body weights and weights of livers, kidneys and ovaries were significantly lower in the groups receiving cytotoxic compounds but not in those treated with D-Trp^6^LH-RH.

In Experiment 3, volume changes of CFPAC-1 tumors are shown in Figure 3 and numerical data are shown in Table 1. Tumor volume was significantly lower in the groups treated with AN-152. Treatment with DOX resulted in a moderate but not significant decrease in tumor volume. Tumor weights were also reduced after treatment with AN-152, but the differences from control were statistically not significant. Body weights were the lowest in the group receiving DOX, but the differences from control were statistically not significant. Organ weights were not changed by the treatments, except for ovarian weights, which were reduced by DOX.

### Gene expression analysis

**Figure 4:**
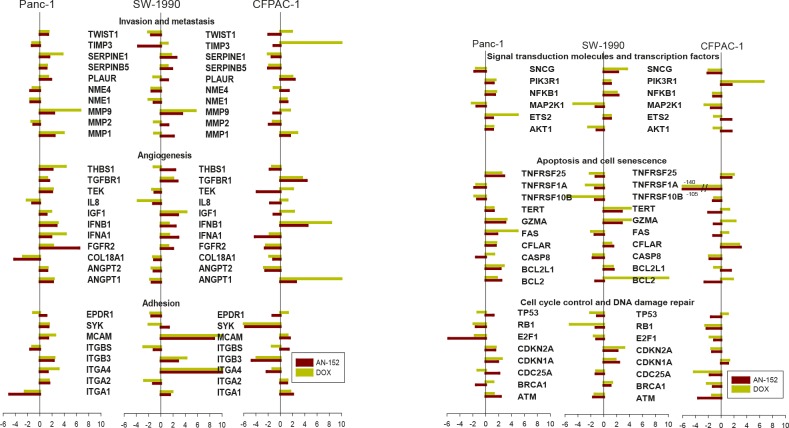
Human pancreatic cancer lines grown in nude mice were analyzed with the Human Cancer Pathway Finder RT Profiler PCR Array The bars show fold changes compared to control. *= P<0.05 vs control. The genes are grouped according to their functions as follows. (A) Invasion and metastasis: TWIST1: Twist homolog 1; TIMP3: TIMP metallopeptidase inhibitor 1; SERPINE1: Serpine peptidase inhibitor, clade E, member 1; SERPINB5: Serpin peptidase inhibitor, clade B, member 5; PLAUR: Plasminogen activator, urokinase receptor; NME4, NME1: Non-metastatic cells protein 4, 1; MMP9, 2, 1: Matrix metallopeptidase 9, 2, 1. Angiogenesis: THBS1: Thrombospondin 1; TGFBR1: Transforming growth factor beta receptor 1; TEK: TEK tyrosine kinase; IL8: Interleukin 8; IGF1: Insulin-like growth factor 1; IFNB1: Interferon, beta 1; IFNA1: Interferon, alpha 1; FGFR2: Fibroblast growth factor receptor 2; COL18A1: Collagen, type XVIII, alpha 1; AGPT2, 1: Angiopoietin 2, 1. Adhesion: EPDR1: Ependymin related protein 1; SYK: Spleen tyrosine kinase; MCAM: Melanoma cell adhesion molecule; ITGB, ITGA: Integrin beta, alpha. (B) Signal transduction molecules and transcription factors: SNCG: Synuclein gamma (breast cancer specific protein); PIK3R1: Phosphoinositide-3-kinase, regulatory subunit 1; MAP2K1: Mitogen-activated protein kinase 1; ETS2: V-Ets erythroblastosis virus E26 oncogene homolog 2; AKT1: V-akt murine thymoma viral oncogene homolog 1. Apoptosis and cell senescence: TNFRSF25, 1A, 10B: Tumor necrosis factor receptor superfamily member 25, 1a, 10b; TERT: Telomerase reverse transcriptase; GZMA: Granzyme A; FAS: Fas (TNF receptor superfamily, member 6; CFLAR: CASP8 and FADD-like apoptosis regulator; CASP8: Caspase 8; BCL2L1: BCL2-like 1; BCL2: B-cell CLL/lymphoma 2. Cell cycle control and DNA damage repair: TP53: Tumor protein p53; RB1: Retinoblastoma 1; E2F1: E2F transcription factor 1; CDKN2A, 1A: Cyclin-dependent kinase inhibitor 2A, 1A; CDC25A: Cell division cycle 25 homolog A; BRCA1: Breast cancer 1; ATM: Ataxia telangiectasia mutated.

To discover differences in the effects of AN-152 and DOX on molecules important in cancer development and progression, the Human Cancer Pathway Finder PCR Array was used. The expression of genes in the treated tumors compared to controls is shown in Fig. 4A,B. Among the genes related to invasion and metastasis, both decreases and increases in expression were observed in all three tumor lines, with DOX causing the stronger increases. Expression of angiogenesis-related genes varied in different directions in the three tumors; mostly increases were seen in Panc-1 and SW-1990 while mostly decreases, in CFPAC-1. The decreases were caused mostly by AN-152 and the increases by DOX. Expression of genes associated with adhesion was decreased in Panc-1 and CFPAC-1 cancers by both compounds, somewhat more strongly by AN-152, and were changed in both directions by both agents in SW-1990 tumors (all Fig. 4A). Both AN-152 and DOX caused changes in expression of the genes related to signal transduction molecules and transcription factors; DOX caused considerable increases or decreases in some. Increases and decreases in expression of apoptosis-associated genes were variable in the three tumors after both treatments. The genes associated with cell cycle control and DNA damage repair were altered in both directions in Panc-1 and SW-1990 cancers, and showed only decreases in CFPAC-1 cancers (all Fig. 4B).

**Figure 5:**
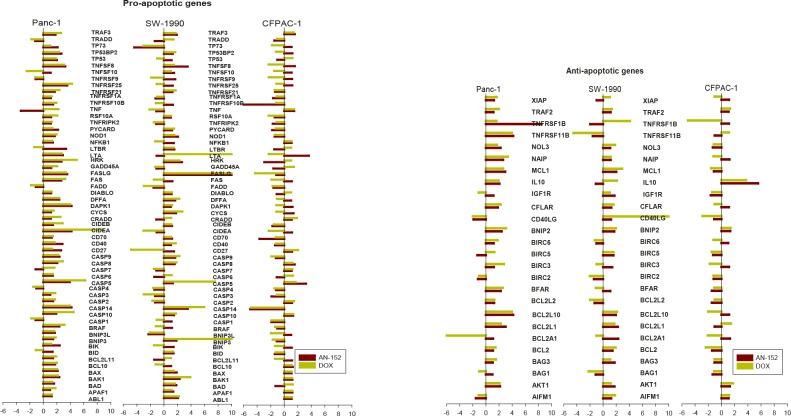
Human pancreatic cancer lines grown in nude mice were analyzed with the Human Apoptosis RT Profiler PCR Array as described in the Materials and Methods The vertical bars show fold changes in gene expression compared to control. *= P<0.05 vs control. (A) Pro-apoptotic genes: TRAF3: TNF receptor-associated factor 3; TRADD: TNFRSF1A-assiciated via death domain; TP73: Tumor protein p73; TP53BP2: Tumor protein p53 binding protein 2; TP53: Tumor protein p53; TNFSF8, 10: Tumor necrosis factor superfamily, member 8, 10; TNFRSF10, 9 25, 21, 1A, 10A, 10B: Tumor necrosis factor receptor superfamily, member 10, 9, 25, 21, 1A, 10A, 10B; TNF: Tumor necrosis factor; RIPK2: Receptor-interacting serine-threonine kinase 2; PYCARD: PYD and CARD domain containing; NOD1: Nucleotide-binding oligomerization domain containing 1; NFKB1: Nuclear factor of kappa light polypeptide gene enhancer in B cells 1; LTBR: Lymphotoxin beta receptor; LTA: Lymphotoxin alpha; HRK: Harakiri, BCL2 interacting protein; GADD45A: Growth arrest and DNA-damage-inducible, alpha; FASLG: Fas ligand; FAS: Fas (TNF receptor superfamily, member 6); FADD: Fas-associated via death domain; DIABLO: Diablo, IAP-binding mitochondrial protein; DFFA: DNA fragmentation factor; DAPK1: Death-associated protein kinase 1; CYCS: Cytochrome c, somatic; CRADD: CASP2 and RIPK1 domain containing adaptor with death domain; CIDEB, A: Cell death-inducing DFFA-like effector b, a; CD70, 40, 27: CD70, 40, 27 molecule; CASP: Caspase, apoptosis-related cystine peptidase; BRAF: V-raf murine sarcoma viral oncogene homolog B1; BNIP3L: BCL2, adenovirus E1B 19kDa interacting protein 3-like; BIK: BCL2-interacting killer; BID: BH3 interacting domain death agonist; BCL2L11: BCL2-like 11; BCL10: B-cell CLL/lymphoma 10; BAX: BCL2-associated X protein; BAK1: BCL2-antagonist/killer 1; BAD: BCL2-associated agonist of cell death; APAF1: Apoptotic peptidase activating factor 1; ABL1: C-abl oncogene 1. (B) XIAP: X-linked inhibitor of apoptosis; TRAF2: TNF receptor associated factor 2; TNFRSF1B, 11B: Tumor necrosis factor receptor superfamily member 1B, 11B; NOL3: Nucleolar protein 3; NAIP: NLR family, apoptosis inhibitory protein; MCL1: Myeloid cell leukemia sequence 1; IL10: Interleukin 10; IGF1R: Insulin-like growth factor 1 receptor; CFLAR: CASP8 and FADD-like apoptosis regulator; CD40LG: CD40 ligand; BNIP2: BCL2/adenovirus E1B 19kDa interacting protein 2; BIRC6, 5, 3, 2: Baculoviral IAP repeat containing 6, 5, 3, 2; BFAR: Bifunctional apoptosis regulator; BCL2L2, 10, 1, A1: BCL2-like…; BCL2: B-cell CLL/lymphoma 2; BAG3, 1: BCL2-associated athanogene 3, 1; AKT1: V-akt murine thymoma viral oncogene homolog 1; AIFM1: Apoptosis-inducing factor, mitochondrium-associated, 1.

To analyze the effects of AN-152 and DOX on expression of apoptosis-related genes, the Human Apoptosis RT Profiler PCR Array was used. Among the pro-apoptotic genes investigated, several were increased in Panc-1 and decreased in CFPAC-1 cancers, but were changed in both directions in SW-1990 (Fig. 5A). Thus, the expression of anti-apoptotic genes was mostly increased in Panc-1 and SW-1990 and decreased in CFPAC-1 tumors, similarly by both agents (Fig. 5B).

## DISCUSSION

Recent advances in understanding the molecular mechanisms involved in the genesis and progression of pancreatic cancer have provided us with novel therapeutic approaches. Among the new modalities, which have become commercially available, some target growth factors and their receptors, (e.g. EGF or PDGF) and intracellular kinases (e.g. protein kinase C), and some target pro-angiogenic molecules (e.g. VEGF or interleukin-8). [14, 32-34]

Receptors for various peptide hormones are present in pancreatic cancers and these receptors can be utilized for both diagnostic and therapeutic purposes. [15, 21]. LH-RH receptors in experimental and human pancreatic tumors were demonstrated long ago. [15, 21] however, their function and importance is still unclear. The combined detection of mRNA for both LH-RH and LH-RH receptor in rat pancreatic cancer lines raised the possibility of an autocrine/paracrine role of LH-RH in these tumors. [35] The findings that both estrogen and androgen receptors are likewise expressed in pancreatic carcinoma indicate that sex steroids may also play a role in the growth of these tumors. We have shown in several experiments that treatment with an LH-RH agonist, D-Trp^6^LH-RH, or antagonist, cetrorelix, inhibits growth of nitrosamine-induced pancreatic cancers in hamsters. [15, 21] This inhibition can be the result of the blockade of the pituitary-gonadal axis or of a direct effect through LH-RH receptors in tumors. Unfortunately, clinical trials with LH-RH agonists have had disappointing results in pancreatic cancer patients. [15, 21] The actual mechanism of action of cytotoxic LH-RH analogs differs from those of hormone agonists or antagonists however, as in this situation the hormone performs only a delivery role, carrying the cytotoxic moiety to tumor cells that express specific receptors for that hormone. Thus, AN-152 (AEZS-108) acts primarily as a cytotoxic molecule targeted to tumor cells rather than as a hormonal agent. The inhibitory effect of AN-152 (AEZS-108) on two pancreatic cancer lines was recently demonstrated by others. [36]

In the present study we demonstrated the presence of LH-RH receptors in clinical pancreatic cancer samples as well as in three human pancreatic cancer cell lines. Cytotoxic LH-RH analog, AN-152 (AEZS-108) powerfully inhibited growth of all three of these lines xenografted into nude mice. DOX alone also had an inhibitory effect on growth of SW-1990 tumors, but AN-152 acted significantly more strongly than DOX on Panc-1 and CFPAC-1 tumors.

In previous studies, various other of our cytotoxic conjugates, such as cytotoxic analog of LH-RH (AN-207), somatostatin (AN-238) and bombesin (AN-215), powerfully inhibited growth in nude mice of human pancreatic cancer lines that expressed the specific receptors for those carrier peptides (17, 18). Herein, we demonstrated that cytotoxic LH-RH analog, AN-152 (AEZS-108), also has a strong inhibitory effect on experimental pancreatic cancers.

PCR arrays have been successfully used in our previous studies to detect molecular changes elucidating the effects of our antineoplastic compounds. [37-39] Using the PCR array method in the present study, we also discovered some differences between the effects of the cytotoxic hormone analog and the cytotoxic radical moiety. The genes which were investigated encode proteins that are involved in various biological pathways important in tumorigenesis and tumor progression. The changes were each relatively modest, probably because the animals were sacrificed on the average of 3 weeks after the last injection. Small changes add up however, as many genes were affected. Differences between the effects of AN-152 and the DOX moiety were demonstrated mainly in genes associated with invasion and metastasis, adhesion and angiogenesis. AN-152 affected MMP-related genes more markedly than DOX; however, DOX strongly increased TIMP3, which is an MMP inhibitor. Metalloproteinases are responsible for extracellular matrix and basement membrane degradation in tumors and thus have a major role in invasion and metastasis. MMP1, MMP2 and MMP9, as well as their inhibitors, TIMP1 and TIMP2, were detected in pancreatic cancers, and their concentrations correlated with tumor grade, tumor size, invasive characteristics, and metastasis. [40-42] Drugs that inhibit the activity of MMPs in cancer progression have been sought, developed and tested clinically, thus the ability of AN-152 to decrease MMP levels may be an important factor in its mechanism of tumor inhibition, and its efficacy.

Both AN-152 and DOX decreased expression of genes connected to adhesion in Panc-1 and CFPAC-1 tumors, with AN-152 having a slightly stronger effect. In CFPAC-1 cancers, angiogenesis-related gene expression was strongly decreased by AN-152 and strongly increased by DOX. There was no major difference in the effects of AN-152 and DOX on other gene expression categories, including apoptosis-related genes. Consequently, it is obvious that these effects depend not only on the compounds used for therapy, but also on the type of tumor. Thus, it is understandable that in these experiments with pancreatic tumors the two compounds similarly affected genes associated with apoptosis, while they acted differently on these same genes in bladder cancers [43] or glioblastomas [44] in other experiments.

Summarizing the results of our study, we can state that receptors for LH-RH are expressed in human pancreatic cancers, and that receptor-targeted therapy with the cytotoxic LH-RH analog, AN-152 (AEZS-108), has a stronger inhibitory effect on the growth of human pancreatic cancer lines in nude mice than the cytotoxic radical, DOX. In SW-1990 cancers, where LH-RH receptor expression seemed to be the lowest, the difference between the effects of the two compounds was the least. Because of the poor response to conventional chemotherapy, radiation therapy, and surgery, patients with any stage of pancreatic cancer can appropriately be considered as candidates for clinical trials with this agent. Based on our results, AEZS-108 (AN-152) deserves to be tried for treatment of patients with LH-RH receptor positive pancreatic cancers.

## MATERIALS AND METHODS

### Ethics Statement

This investigation has been conducted in accordance with the ethical standards and according to the Declaration of Helsinki and according to national and international guidelines and has been approved by the authors' institutional review board.

### Detection of LH-RH receptors in human pancreatic cancer specimens

Two archived samples of surgical specimens of human pancreatic cancers were used for the immunohistochemical studies. These were formalin-fixed and paraffin-embedded. Three micrometer thick sections were stained with hematoxylin and eosin to confirm the presence of pancreatic adenocarcinoma. Adjacent serial sections were then utilized for the immunoperoxidase procedure following standard protocol. Briefly, the slides were deparaffinized, rehydrated and washed in phosphate-buffered saline solution. Antigen retrieval was performed in a pressure cooker with Dako Target Retrieval Solution (S1968; Dako, Carpinteria, CA) at 90˚C for 15 min (pH 9.0). Antibodies to LH-RH receptor (NCL-GnRHR A9E4; Novocastra Laboratories Ltd, Newcastle upon Tyne , United Kingdom and GnRHR-N20, Santa Cruz Biotechnology, Santa Cruz, CA) were added and slides incubated for 30 min at room temperature. NCL-GnRHR is a monoclonal antibody raised in mouse and targets the human LH-RH receptor extracellular region. The GnRH-R N20 is an affinity-purified goat polyclonal antibody and maps near the N-terminus of human LH-RH-R. Following the addition of the detection system, the reaction was visualized using diaminobenzidine. The slides were counterstained with hematoxylin. Human pituitary (anterior lobe), obtained from autopsy, was used as positive control.

### Materials

LH-RH agonist [D-Lys^6^]LH-RH (pyroGlu-His-Trp-Ser-Tyr-D-Lys-Arg-Pro-Gly-NH_2_) carrier was synthesized in our laboratory by solid-phase methods. DOX^.^HCl salt was purchased from Chemex Export-Import GmbH (Vienna, Austria). Cytotoxic LH-RH conjugate AN-152 was synthesized by AEterna/Zentaris (Frankfurt am Main, Germany). Chemicals, unless stated otherwise, were purchased from Sigma (St. Louis, MO). For treatment, the cytotoxic compounds were dissolved in 0.01 M aqueous acetic acid and diluted with 5.5% (w/v) aqueous D-mannitol, and 0.2 ml/20 g body weight of this solution was injected into the jugular veins of the test mice.

### Animals and Tumors

Female athymic nude mice (Ncr nu/nu) were obtained from the National Cancer Institute (Frederick Cancer Research and Development Center, Frederick, MD), Charles River Laboratories International, Inc. (Durham, NC) and Harlan Laboratories (Tampa, FL) and maintained under pathogen-limited conditions. SW-1990, Panc-1 and CAPAN-1cells were purchased from the American Type Culture Collection (ATCC; Manassas, VA) and maintained in culture according to ATCC's recommendation. To produce tumor donor animals, 10^6^ cells of each line were injected subcutaneously into 3 nude mice and grown until well-developed tumors were grown. The resulting tumors were removed, minced and 2 mm^3^ pieces of tumor tissue were transplanted subcutaneously into each flank area of the experimental nude mice. When the tumors became measurable, the mice were divided into groups with nearly equal average tumor sizes. Control and treatment groups were then selected at random.

### Experimental protocol

In Experiment 1, nude mice with Panc-1 tumors were treated iv. on days 1, 8. 15, 22, 33 and 61 as follows: Group 1: Control (vehicle only); Group 2: AN-152, 6.9 μmol/kg; Group 3, DOX, 2.5 μmol/kg. There were 8 animals in each group. The experiment was terminated on day 82.

In Experiment 2, the treatment of mice with SW-1990 cancers started 33 days after implantation as follows: Group 1: Control; Group 2: AN-152; Group 3: DOX, Group 4: D-Trp^6^[LH-RH]. All compounds were administered iv.(at 6.9 μmol/kg) once weekly for 7 weeks. There were 10 animals per group. The experiment was terminated 65 days after the first treatment.

In experiment 3, the treatment started 87 days after implantation of CFPAC-1 cancers as follows: Group 1: Control; Group 2: AN-152; Group 3: DOX. The compounds were given iv. on days 1, 5, 8, 12, 15 and 30. There were 8 animals per group, and the experiment lasted to the 61st day of the treatment period.

### Receptor binding assay

LH-RH receptors were analyzed by the binding of ^125^I-labeled [D-Trp^6^]LH-RH to tumor membrane homogenates of control animals as described. [45] Four analyses were done in all samples in duplicate or triplicate tubes and binding characteristics were acquired from 12-point displacement experiments. The LIGAND PC computerized curve-fitting program was used to determine the type of binding, the maximal binding capacity of the receptors and the dissociation constant (31).

### Molecular analysis

After isolating total RNA and protein from tumor samples from experiments 2, 3 and 4 by using the NucleoSpin kit (Macherey-Nagel, Bethlehem, PA, USA), the Human Cancer Pathway Finder Superarray and the Human Apoptosis RT Profiler PCR Array (Qiagen Inc. Valencia, CA, USA) were used to analyze 3 representative samples from each group for mRNA levels of genes related to cell adhesion, apoptosis, cell cycle, angiogenesis, signal transduction, invasion and metastasis. Quality control of RNA samples, synthesis of cDNA and RT-PCR arrays were performed as described. [46] Gene expression data were examined using Excel based PCR Array Data Analysis Software provided by Qiagen; fold-changes in gene expression were calculated using the ΔΔCt method and five housekeeping genes (B2M, HPRT1, RPL13A, GAPDH, and ACTB) were used for normalization of the results.

The proteins isolated from three tumor samples in Experiments 2, 3 and 4 were processed for Western blotting to detect expression of LH-RH receptors as described. [47] Briefly, isolated proteins were sonicated and the lysates were adjusted to equal concentrations. Primary antibodies for LH-RH receptor were purchased from Abcam (ab-58561; Cambridge, MA, USA). The immunoreactive bands were visualized with the Odyssey Infrared Imaging System, and 3.0 software was used (LI-COR Biosciences, Lincoln, NE, USA).

### Statistical analysis

One-way ANOVA (Sigmaplot-11 software; Jandel Scientific, San Raphael, CA) was used for statistical evaluation of the data, and the groups were compared using Dunnett's method.
